# Transpulmonary thermodilution-derived haemodynamics in patients with liver failure: a prospective study in 351 patients

**DOI:** 10.1186/cc14249

**Published:** 2015-03-16

**Authors:** W Huber, A Breitling, B Henschel, S Mair, S Goetz, J Tschirdewahn, J Frank, A Beitz, RM Schmid

**Affiliations:** 1 Klinikum rechts der Isar, Technical University of Munich, Germany

## Introduction

Patients with acute or chronic liver failure are considered to have an altered pattern of haemodynamics. Nevertheless, there is a lack of studies systematically investigating haemodynamics in patients with liver failure. Therefore, it was the aim of this study to compare transpulmonary thermodilution (TPTD)-derived haemodynamics of 112 patients with acute or chronic liver failure with 239 patients without liver failure.

## Methods

We analyzed a prospectively maintained database including 6,016 TPTD measurements in 351 patients. To account for different numbers of TPTDs in different patients, comparison of first measurements of patients with and without liver failure was the primary endpoint. Statistics: Wilcoxon test for unpaired samples; IBM SPSS Statistics 22.

## Results

A total of 207 male and 144 female patients, APACHE II score 21 ± 7, 62 ± 14 years old, one to 126 TPTDs per patient. Diagnosis: cirrhosis/liver failure *n *= 112 patients (31.9%), sepsis 55 (15.7%), ARDS 46 (13.1%), GI affection 21 (6.0%), cardiogenic shock 19 (5.4%), various 98 (27.9%). Patients with liver failure were slightly younger than the other patients (58 ± 11 vs. 64 ± 15 years; *P *< 0.001). All other baseline characteristics were comparable including APACHE II (20 ± 7 vs. 21 ± 8; NS), SAPS (39 ± 12 vs. 41 ± 14; NS), height (172 ± 9 vs. 170 ± 9 cm; NS) and weight (76 ± 20 vs. 73 ± 17 kg; NS). Among haemodynamic parameters, preload markers GEDVI (753 ± 168 vs. 790 ± 226 ml/m^2^; *P *= 0.182) and CVP (14.4 ± 8.8 vs. 14.9 ± 7.1 mmHg; *P *= 0.250) were comparable. Despite comparable preload parameters, the following parameters were significantly different: patients with acute or chronic liver failure had significantly higher cardiac index (4.3 ± 1.3 vs. 3.3 ± 1.3 l/minute/ m^2^; *P *< 0.001), stroke volume index (50 ± 15 vs. 37 ± 15; *P *< 0.001), pulse pressure (75 ± 19 vs. 65 ± 21 mmHg; *P *= 0.021) and cardiac power index (0.7 ± 0.24 vs. 0.60 ± 0.28 W/m^2^; *P *< 0.001). By contrast, MAP (77 ± 15 vs. 80 ± 15 mmHg; *P *= 0.045), SVRI (1,305 ± 638 vs. 1,877 ± 898 dyn*s/ cm^5^*m^2^; *P *< 0.001) and heart rate (84 ± 19 vs. 92 ± 22/minute; *P *< 0.001) were significantly lower in patients with liver failure.

## Conclusion

Our data derived from a large TPTD database demonstrate markedly different haemodynamics in patients with cirrhosis or acute liver failure with the only exception of static preload markers GEDVI and CVP. These findings should be considered in instable patients with liver failure.

